# Resolving primary pathomechanisms driving idiopathic-like spinal curvature using a new *katnb1* scoliosis model

**DOI:** 10.1016/j.isci.2022.105028

**Published:** 2022-08-28

**Authors:** Anne Meyer-Miner, Jenica L.M. Van Gennip, Katrin Henke, Matthew P. Harris, Brian Ciruna

**Affiliations:** 1 Program in Developmental & Stem Cell Biology, The Hospital for Sick Children, 686 Bay Street, Toronto, ON M5G 0A4, Canada; 2 Department of Molecular Genetics, The University of Toronto, Toronto, ON M5S 1A8, Canada; 3 Department of Orthopedic Research, Boston Children’s Hospital, Department of Genetics, Harvard Medical School, Boston, MA 02115, USA; 4 Department of Orthopaedics and Department of Human Genetics, Emory University, Atlanta, GA 30322, USA

**Keywords:** Molecular genetics, Molecular biology experimental approach, Model organism

## Abstract

Idiopathic scoliosis (IS) refers to abnormal spinal curvatures that occur in the absence of vertebral or neuromuscular defects. IS accounts for 80% of human spinal deformity, afflicts ∼3% of children worldwide, yet pathogenic mechanisms are poorly understood. A key role for cerebrospinal fluid (CSF) homeostasis in zebrafish spine development has been identified. Specifically, defects in cilia motility of brain ependymal cells (EC), CSF flow, and/or Reissner fiber (RF) assembly are observed to induce neuroinflammation, oxidative stress, abnormal CSF-contacting neuron activity, and urotensin peptide expression, all associating with scoliosis. However, the functional relevance of these observations to IS remains unclear. Here we characterize zebrafish *katnb1* mutants as a new IS model. We define essential roles for Katnb1 in motile ciliated lineages, uncouple EC cilia and RF formation defects from spinal curvature, and identify abnormal CSF flow and cell stress responses as shared pathogenic signatures associated with scoliosis across diverse zebrafish models.

## Highlights

• Characterize *katnb1* mutant zebrafish as a new model of idiopathic scoliosis (IS)
• Define critical role for Katnb1 in motile ciliated lineages, including choroid plexus
• Uncouple ependymal cell cilia and Reissner fiber formation defects from spinal curves
• Identify abnormal CSF flow and cell stress response as common signatures linked to IS

## Graphical abstract

**Figure:**
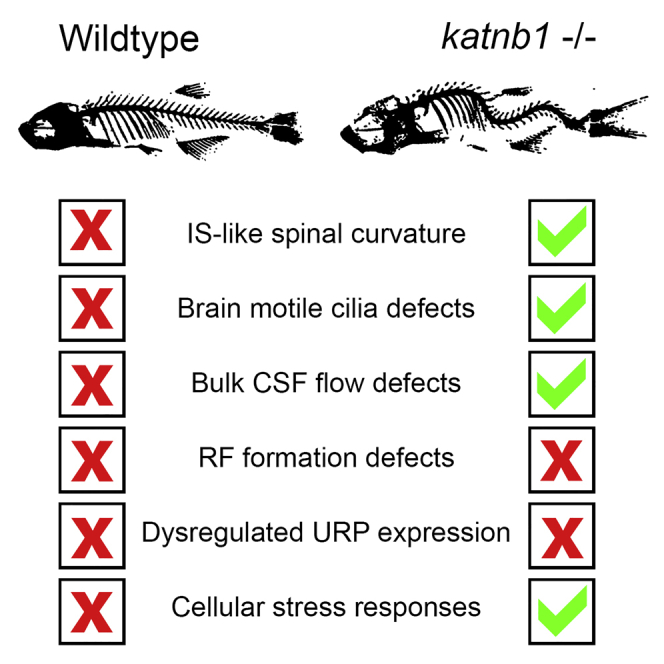

## Introduction

Abnormal curvatures of the spine, known as scoliosis, have been pervasive throughout human history (Naderi et al., 2007; Vasiliadis et al., 2009). The most prevalent type, idiopathic scoliosis (IS), manifests as a rotational spinal curvature that develops in the absence of gross vertebral malformations or other physiological defects, often during periods of intense growth linked to adolescence (Horne et al., 2014; Cheng et al., 2015). IS afflicts 3%–4% of school-aged children, yet our understanding of pathogenic mechanisms underlying IS remains clouded in large part by its genetic heterogeneity (Kesling and Reinker, 1997; Wise et al., 2008; Cheng et al., 2015; Grauers et al., 2016; Peng et al., 2020). Recent whole exome sequencing (WES) studies and multi-ethnic GWAS meta-analyses have associated variants in musculoskeletal collagen and cartilaginous extracellular matrix genes with IS susceptibility for a subset of cases (Haller et al., 2016; Khanshour et al., 2018). However, it is estimated that >95% of the total genetic variance underlying IS remains to be discovered (Kou et al., 2019). This fact underscores a critical need to better understand IS pathobiology.

Surprisingly, spinal curvatures rank among the most common deformities observed in teleost fish, and utilization of zebrafish genetic IS models has spearheaded efforts across multiple research groups to elucidate the biological underpinnings of spinal curve progression (Hayes et al., 2013, 2014; Grimes et al., 2016; Van Gennip, Boswell and Ciruna, 2018; Zhang et al., 2018; Rose et al., 2020; Terhune et al., 2020; Troutwine et al., 2020; Gray et al., 2021; Wang et al., 2022). Pioneering work with zebrafish *protein tyrosine kinase 7a* (*ptk7a*) mutants characterized the first genetically defined developmental model of IS and identified a key role for cerebrospinal fluid (CSF) homeostasis in spine development, linking motile cilia-driven CSF flow defects with idiopathic-like spinal curvatures (Hayes et al., 2013, 2014; Grimes et al., 2016; Van Gennip, Boswell and Ciruna, 2018). Notably, there is some evidence that cilia and CSF flow defects may also be linked to human IS (Patten et al., 2015; Oliazadeh et al., 2017; Baschal et al., 2018; Terhune et al., 2020; Wang et al., 2020; Algin et al., 2022).

Recently, zebrafish *scospondin* (*sspo*) mutants were identified to develop idiopathic-like scoliosis in the absence of cilia abnormalities (Rose et al., 2020; Troutwine et al., 2020). Rather, irregularities in CSF flow associated with the disrupted formation of Reissner fiber (RF, a proteinaceous filament that threads through ventricles of the brain and spinal cord) were linked to *sspo* IS phenotypes. However, as defects in bulk CSF flow and RF aggregation are shared across *sspo*, *ptk7a*, and other zebrafish IS models (Rose et al., 2020; Troutwine et al., 2020), the primary cause of scoliosis remains unclear. Downstream of CSF homeostasis and RF deficits, abnormal CSF-contacting neuron (CSF-CN) activity, urotensin neuropeptide (Urp) expression, neuroinflammatory signals, and oxidative stress have all been associated with axial curvature (Böhm et al., 2016; Cantaut-Belarif et al., 2018, 2020; Sternberg et al., 2018; Van Gennip, Boswell and Ciruna, 2018; Zhang et al., 2018; Lu et al., 2020; Orts-Del’Immagine et al., 2020; Rose et al., 2020). The interdependence and functional relation of these phenotypes to idiopathic-like scoliosis remains to be determined.

To gain further insights into the etiopathogeneses of spinal curvature, we characterize zygotic *katanin p80 subunit* mutants (*katnb1*^*mh102/mh102*^) (Hu et al., 2014) as a new zebrafish IS model. We define essential roles for Katnb1 in motile cilia and choroid plexus (ChP) development. Through this analysis, *katnb1* mutants functionally uncouple brain ependymal cell (EC) cilia, *urp* expression, and RF formation defects from spinal curvature. Rather, our studies identify physiological stress responses downstream of CSF homeostasis defects as biological mechanisms associated with idiopathic-like scoliosis that are shared across diverse zebrafish IS models.

## Results

### *katnb1* mutants develop late-onset spinal curvatures that model human IS

Katanin is a microtubule severing heterodimer comprised of catalytic p60 and scaffolding p80 subunits, with the p80 subunit (KATNB1) functioning to localize Katanin to the centrosome (McNally et al., 1996; Hartman et al., 1998). In single-celled organisms and in vertebrates, Katanin has been found to play essential roles in flagella motility and ciliogenesis (Dymek et al., 2004; Sharma et al., 2007; Mirvis et al., 2019). Based on human *KATNB1* mutations linked to microlissencephaly, studies utilizing cell culture and mouse models also revealed the functional importance of KATNB1 in centriole biogenesis, Shh signaling, and neural progenitor development (Hu et al., 2014; Mishra-Gorur et al., 2014), whereas analysis of maternal-zygotic (MZ) *katnb1* mutant zebrafish demonstrated a critical role for Katnb1 in early embryonic patterning and viability (Hu et al., 2014).

**Figure 1:**
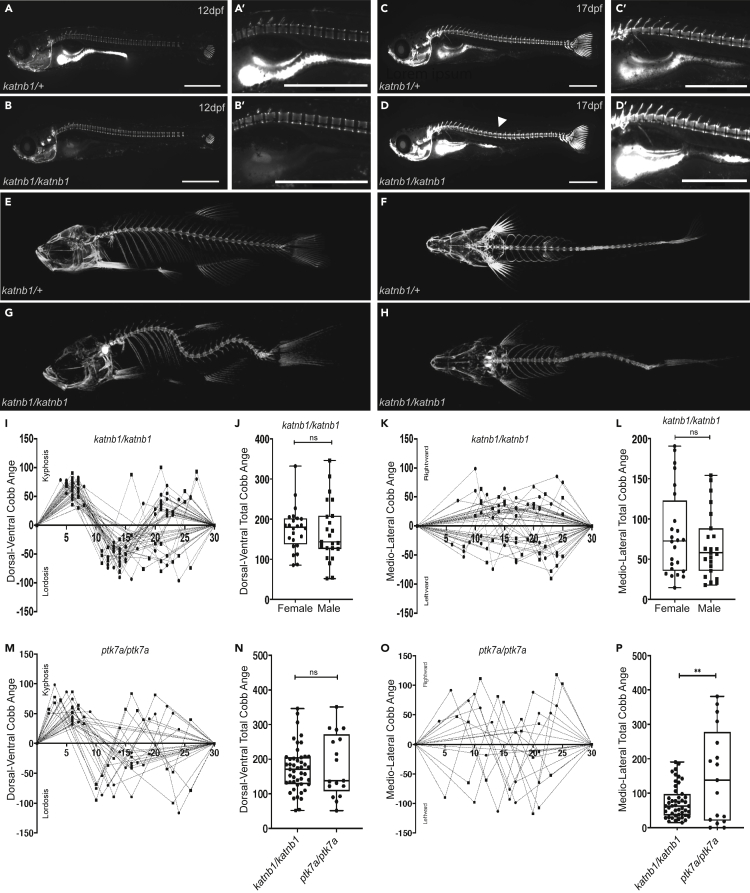
*katnb1* mutant zebrafish exhibit defining attributes of IS (A–D) Representative lateral views of calcein-stained *katnb1*^*mh102/+*^ (A & A’; N = 2, n = 24) and *katnb1*^*mh102/mh102*^ (B & B’; N = 2, n = 13) fish at 12 dpf (6 mm average length); and of *katnb1*^*mh102/+*^ (C & C’; N = 4, n = 53) and *katnb1*^*mh102/mh102*^ (D & D’; N = 4, n = 18) fish at 17 dpf (7 mm average length). Note onset of spinal curvatures in *katnb1*^*mh102/mh102*^ zebrafish (arrowheads) in the absence of congenital vertebral malformations. Squares indicate location of higher magnification images (A′–D′). Scale bars, 1 mm. (E–H) Three dimensional microCT projections of representative 3-month-old *katnb1*^*mh102/+*^ (E & F; N = 2, n = 5) and *katnb1*^*mh102/mh102*^ (G & H; N = 17, n = 68) zebrafish viewed in sagittal (E & G) and coronal (F & H) planes. (I–L) Quantification of curve severity, direction, and position along the DV (I, J) and ML (K, L) axes of *katnb1*^*mh102/mh102*^ mutant zebrafish. (I, K) Graphs depicting spinal curvatures of individual mutant fish: x axis indicates vertebral position of curve apices along the rostral to caudal plane; Y axis indicates magnitude of curvature (Cobb angle). Positive Cobb angles represents kyphotic curves in the DV axis and rightward curves in the ML axis. Negative Cobb angle represents lordotic curves in the DV axis and leftward curves in the ML axis. (J, L) Graphs depicting sums of all Cobb angle measurements in DV (J, p = 0.72) and ML (L, p = 0.31) axes for male and female *katnb1*^*mh102/mh102*^ fish (N = 6, n = 47). Statistical analyses were performed using student’s t test. (M and O) Quantification of curve severity, direction, and position along the DV (M) and ML (O) axes of *ptk7a*^*hsc9/hsc9*^ mutant zebrafish. (N and P) Quantification of curve severity as a measure of total Cobb angle, comparing *katnb1*^*mh102/mh102*^ (N = 17, n = 68) and *ptk7a*^*hsc9/hsc9*^ (N = 8, n = 17) mutant animals along the DV (N, p = 0.613) and ML (P, p = 0.0014) axis.

In contrast to strong MZ *katnb1* mutant phenotypes, zygotic *katnb1*^*mh102/mh102*^ mutant zebrafish display no overt morphological abnormalities at embryonic stages, owing to maternally contributed RNAs (Hu et al., 2014). However, at late larval and early juvenile stages (7–8 mm standard length; approximately 3 weeks post fertilization [wpf]), we observed that zygotic *katnb1*^*mh102/mh102*^ mutants develop obvious axial curvatures (Figure 1). In order to characterize vertebrae formation, we used the vital dye calcein, which binds to calcified skeletal structures (Du et al., 2001), and observed no obvious vertebral segmentation defects prior to or post-onset of curve formation (Figures 1A–1D). Axial curvatures emerge only during late juvenile stages of development and could first be detected in some *katnb1*^*mh102/mh102*^ mutants at 17 dpf (days postfertilization; ∼7 mm length; Figures 1D and 1D′). The majority of mutants develop curves between 21 and 23 dpf, and scoliosis is fully penetrant by 30 dpf.

To better visualize spine deformities, we performed microcomputed tomography (μCT) of 3-month-old (3mo) adults (Figures 1E–1H) and quantified the position, magnitude, and orientation of spinal curvatures (Figure S1). Within the dorsal-ventral (DV) or sagittal plane, the position and orientation of curves are well conserved along the rostral-caudal axis of *katnb1*^*mh102/mh102*^ mutants (Figures 1G and 1I), beginning with a stereotypic kyphotic curvature correlating with the positioning of the swim bladder (Parichy et al., 2009) followed by a compensatory lordotic curve in the opposite direction. The most caudal spinal curvatures in *katnb1*^*mh102/mh102*^ mutants are more variable in nature, perhaps free from biomechanical constraints of underlying organ systems.

Spinal curvatures in the medio-lateral (ML) or coronal plane are less severe than sagittal curves and vary in both their position and left versus right chirality along the rostral-caudal axis (Figures 1H and 1K). Although no significant difference in scoliosis severity was observed between male and female *katnb1*^*mh102/mh102*^ mutants, cumulative spinal curve measurements trended higher in females (Figures 1J and 1L). Overall, *katnb1*^*mh102/mh102*^ mutant phenotypes recapitulate many hallmarks of human IS, including an adolescent age of onset, scoliosis progression in the absence of obvious congenital vertebral malformations, and characteristic patterns of spinal curvature. Although the shape and positioning of idiopathic-like curves differ between human IS patients and zebrafish *katnb1* mutant models, curve patterns likely reflect biomechanical forces imposed by species-specific organ anatomy (Schlösser et al., 2017).

### *katnb1* scoliosis phenotypes are associated with motile cilia and CSF flow defects

**Figure 2:**
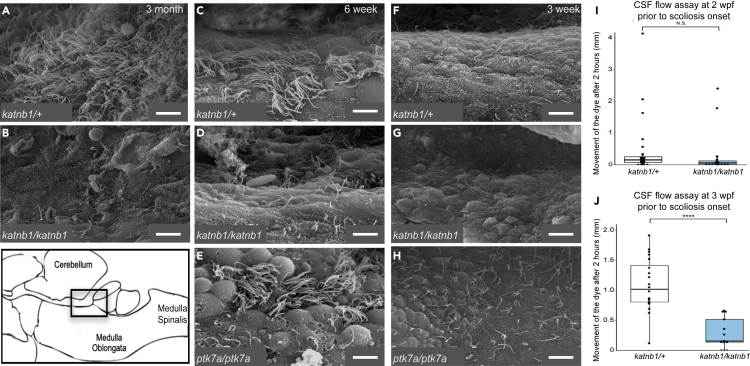
*katnb1* mutants exhibit cilia defects in *foxj1*-positive cell lineages (A–H) SEM imaging of the rhombencephalic ventricle in brains dissected from 3-month-old *katnb1*^*mh102/+*^ (A; N = 3, n = 9) and *katnb1*^*mh102/mh102*^ (B; N = 4, n = 11) adults; 6-week-old *katnb1*^*mh102/+*^ (C; N = 3, n = 9) *katnb1*^*mh102/mh102*^ (D; N = 3, n = 8) and *ptk7a*^*hsc9/hsc9*^ (E; N = 3, n = 9) fish; and 3-week-old *katnb1*^*mh102/+*^ (F; N = 4, n = 14), *katnb1*^*mh102/mh102*^ (G; N = 4, n = 14) and *ptk7a*^*hsc9/hsc9*^ (H; N = 3, n = 8) juveniles. Scale bars, 5 μm. (I and J) Quantification of bulk CSF movement as measured by the distance fluorescent dye travels along the spinal canal (in millimeters; mm), 2 h post-injection into the brain ventricles of experimental fish. (I) *katnb1*^*mh102/+*^ (A; N = 3, n = 37) and *katnb1*^*mh102/mh102*^ (A; N = 3, n = 36) zebrafish exhibit no difference in bulk CSF flow rates at 2 weeks of age (p = 0.4828). (J) Significant differences in bulk CSF flow were observed between *katnb1*^*mh102/+*^ (B; N = 2, n = 19) and *katnb1*^*mh102/mh102*^ mutant (B; N = 2, n = 12) fish at 3 weeks of age (p = 0.0000006829). Statistical analysis was performed using a two tailed t test.

Bulk CSF flow defects have previously been associated with scoliosis in both *ptk7a* and *sspo* zebrafish IS models (Grimes et al., 2016; Van Gennip, Boswell and Ciruna, 2018; Rose et al., 2020). To determine whether similar mechanisms underly spine curvature in *katnb1*^*mh102/mh102*^ mutants, we injected fluorescent dye into the brain ventricles of mutant and sibling control fish and monitored CSF flow into the spinal cord. At 2 wpf, prior to scoliosis onset, *katnb1*^*mh102/mh102*^ mutants and sibling controls exhibit similar, slow rates of CSF flow (Figure 2I). However, at 3 wpf, corresponding with onset of spinal curvature, *katnb1*^*mh102/mh102*^ mutants exhibit a significant reduction in CSF flow as evidenced by restricted distribution of dye along the spinal cord 2 h post-injection (Figure 2J). These results indicate that bulk CSF flow defects are conserved across *ptk7a*, *sspo*, and *katnb1* zebrafish IS models.

We next investigated whether *katnb1*^*mh102/mh102*^ mutants also display defects in EC mono- or multi-cilia formation, which are believed to regulate CSF flow and have been linked with scoliosis in *ptk7a*^*hsc9/hsc9*^ and other zebrafish scoliosis models (Grimes et al., 2016; Konjikusic et al., 2018; Olstad et al., 2019; D’Gama et al., 2021; Wang et al., 2022). Scanning electron microscopy (SEM) of brain rhombencephalic ventricles from 3mo control animals revealed a dense, polarized bed of EC multi-cilia (Figure 2A). In contrast, 3mo *katnb1*^*mh102/mh102*^ mutants exhibited a complete lack of multi-cilia as well as most mono-cilia expected to line brain ventricles (Figure 2B). *katnb1*^*mh102/mh102*^ mutants presented with a more severe cilia loss than that described for *ptk7a*^*hsc9/hsc9*^ mutants (Grimes et al., 2016) but did not exhibit hydrocephalus, which often accompanies EC multi-cilia defects and abnormal CSF flow (Abdelhamed et al., 2018; Figures S2A–S2C). Notably, observed cilia defects were tissue and stage dependent. *katnb1*^*mh102/mh102*^ mutant embryos exhibited normal left-right patterning (a trait commonly disrupted in motile cilia mutants [Essner et al., 2005]); normal differentiation of pronephric motile cilia; as well as normal formation, orientation, and motility of spinal cord floor plate cilia (Figure S3).

**Figure 3:**
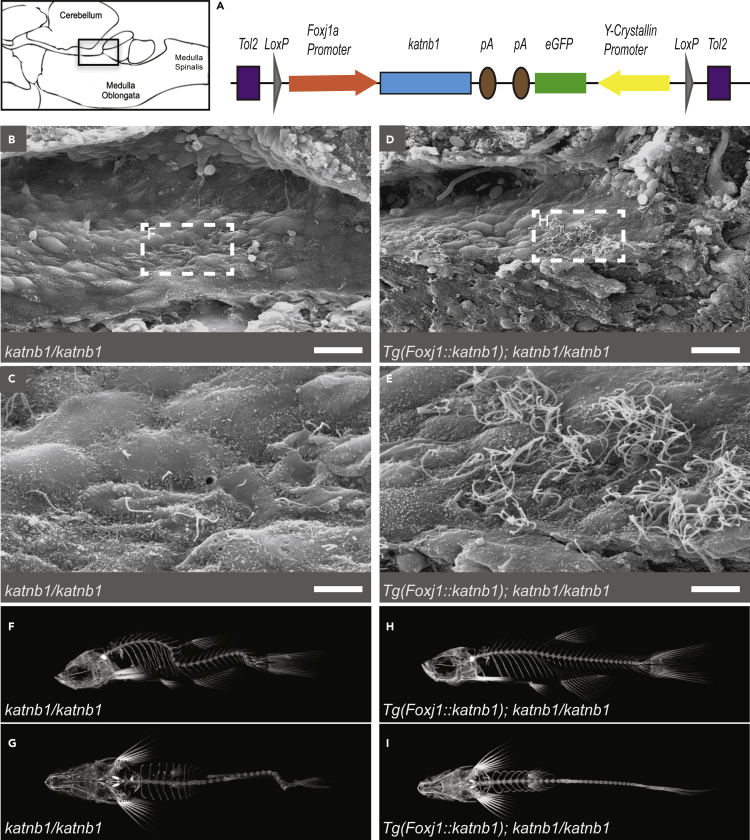
Re-introduction of Katnb1 in *foxj1a*-positive cell lineages suppresses scoliosis (A) Schematic of *Tg(foxj1a*::*katnb1)* construct. (B–E) SEM images of the rhombencephalic ventricle of 3 month old *katnb1*^*mh102/mh102*^ (B & C; N = 4, n = 11) and *Tg(foxj1a*::*katnb1)*; *katnb1*^*mh102/mh102*^ (D & E; N = 4, n = 10) fish. (B, D) Scale bar, 20 μm. Square indicates higher magnification location. (C, E) Scale bar, 5 μm. (F–I) Representative microCT projections of 3-month-old *katnb1*^*mh102/mh102*^ (F & G; N = 17, n = 68) and *Tg(foxj1a*::*katnb1)*; *katnb1*^*mh102/mh102*^ (H & I; N = 8, n = 73) mutant zebrafish in sagittal (F & H) and coronal (G & I) planes.

To determine whether scoliosis specifically results from motile cilia dysfunction, we attempted to suppress spinal curvature in *katnb1*^*mh102/mh102*^ mutants via transgenic re-introduction of wild-type Katnb1 in *foxj1a*-positive motile-ciliated cell lineages (as described in (Grimes et al., 2016); Figure 3A). *Tg(foxj1a*::*katnb1)* expression in *katnb1*^*mh102/mh102*^ mutants resulted in partial suppression of rhombencephalic ventricle EC cilia defects, as imaged by SEM (Figures 3B–3E). Remarkably, *Tg(foxj1a*::*katnb1)* expression fully suppressed scoliosis in *katnb1*^*mh102/mh102*^ mutants (Figures 3F–3I). These results demonstrate that loss of Katnb1 in motile-ciliated lineages induces scoliosis and provide further evidence of a critical role for *foxj1a*-positive cell lineages in spine morphogenesis (Grimes et al., 2016; D’Gama et al., 2021).

### Defects in EC cilia are not specifically linked to spinal curvature

Despite obvious parallels in pathogenic mechanisms associated with scoliosis in *katnb1* and *ptk7a* IS models (CSF flow defects associated with defects in *foxj1a*-positive motile cilia lineages), *katnb1*^*mh102/mh102*^ mutants demonstrate milder scoliosis phenotypes. Quantitative analysis of Cobb angles (a measure of curve severity) revealed that, compared with *ptk7a*^*hsc9/hsc9*^ fish, *katnb1*^*mh102/mh102*^ mutants exhibit a significant reduction in curve severity, specifically in the ML plane (p = 0.0014; Figures 1M−1P). Furthermore, although curve formation in *ptk7a*^*hsc9/hsc9*^ mutants begins around 14 dpf (Hayes et al., 2014; Rose et al., 2020), *katnb1*^*mh102/mh102*^ mutants developed scoliosis later in development, on average by 21 dpf. Thus, *katnb1*^*mh102/mh102*^ animals demonstrate later onset of apparent curvature and less severe spinal curvatures than *ptk7a*^*hsc9/hsc9*^ mutants, despite presenting more severe EC cilia defects at 3 months of age (Grimes et al., 2016; Figures 2A and 2B).

To determine whether phenotypic variances were caused by differences in the timing of EC cilia loss, we performed SEM analysis of rhombencephalic ventricles from *katnb1*^*mh102/mh102*^, *ptk7a*^*hsc9/hsc9*^, and heterozygous control siblings from earlier stages of development. At 6wpf, control fish exhibited well-developed EC multi-cilia throughout the ventricle, although not as dense as 3mo stages (Figure 2C). In contrast, *katnb1*^*mh102/mh102*^ mutants exhibited a clear loss of EC multi-cilia formation at this stage (Figure 2D). Strikingly, *ptk7a*^*hsc9/hsc9*^ mutants exhibited sparse multi-cilia bundles lining the ventricle that appeared similar to control animals (Figure 2E). These results indicate that milder scoliosis phenotypes in *katnb1* versus *ptk7a* mutant zebrafish cannot be explained by later loss of EC cilia in *katnb1*^*mh102/mh102*^ mutants.

Surprisingly, EC multi-cilia had yet to develop in rhombencephalic ventricles of any (mutant or control) animal at 3wpf (Figures 2F–2H). Because *katnb1*^*mh102/mh102*^ and *ptk7a*^*hsc9/hsc9*^ mutants both present nascent spinal curvatures at this stage, this observation supports recent evidence suggesting that loss of EC multi-cilia does not drive spinal curve formation in zebrafish IS models (D’Gama et al., 2021). Instead, given the requirement for Ptk7a and Katnb1 in *foxj1a*-positive cell lineages (Figure 3; Van Gennip, Boswell and Ciruna, 2018), other motile-ciliated cell populations may be functionally responsible to regulate bulk CSF flow and spine morphogenesis.

### *katnb1* mutants exhibit abnormal choroid plexus cilia

*katnb1* expression is enriched in the zebrafish brain (Mishra-Gorur et al., 2014), which exhibits a broad diversity of motile cilia populations (D’Gama et al., 2021). To determine which of these populations may be linked to scoliosis, we performed whole mount immunohistochemistry on 21- and 30-day old *katnb1*^*mh102/mh102*^ mutant versus control brains and screened for alterations in polyglutamylated tubulin (PolyETub) staining, which is a reliable marker for cilia on *foxj1*-expressing cells in zebrafish (Bré et al., 1994; D’Gama et al., 2021).

**Figure 4:**
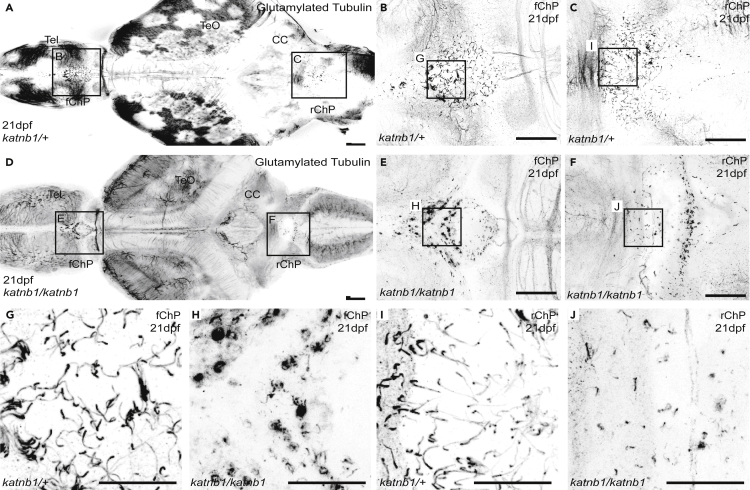
*katnb1* mutants exhibit choroid plexus cilia defects at 21 dpf (A–F) Representative maximum intensity Z-stack projections of confocal micrographs, acquired through dorsally oriented whole mount brains that were dissected from 21dpf *katnb1*^*mh102/+*^ (A; N = 6, n = 14) and *katnb1*^*mh102/mh102*^ (D; N = 6, n = 25) fish, and immunostained for polyglutamylated tubulin. Squares indicate regions of interest, chosen for higher magnification analyses of the forebrain ChP (B & E) and rhombencephalic ChP (C & F) of *katnb1*^*mh102/+*^ control (B & C; N = 8, n = 60) and *katnb1*^*mh102/mh102*^ mutant (E & F; N = 8, n = 32) animals. Scale bars, 50 μm. Tel: telencephalon, TeO: optic tectum, CC: cerebellum. (G–J) Higher magnification regions (as indicated in B, C, E & F) of cilia on the forebrain ChP (G & H) and rhombencephalic ChP (I & J) of *katnb1*^*mh102/+*^ control (G & I) and *katnb1*^*mh102/mh102*^ mutant (H & J) brains. Scale bars, 25 μm.

Notable differences were observed in both the forebrain choroid plexus (fChP) and rhombencephalic choroid plexus (rChP), which are highly ciliated structures found on the dorsal surface of the third and fourth ventricle (in zebrafish) and part of the circumventricular organ (CVO) network that is critical for generating and secreting components of the CSF (Henson et al., 2014; Guerra et al., 2015). In 21 dpf control fish, both ChPs appeared highly ciliated with a prevalence of mono-ciliated cells (Figures 4A–4C, 4G and 4I). However, 21 dpf *katnb1*^*mh102/mh102*^ mutants exhibited lower numbers of ChP mono-cilia (Figures 4D–4F, 4H, and 4J).

**Figure 5:**
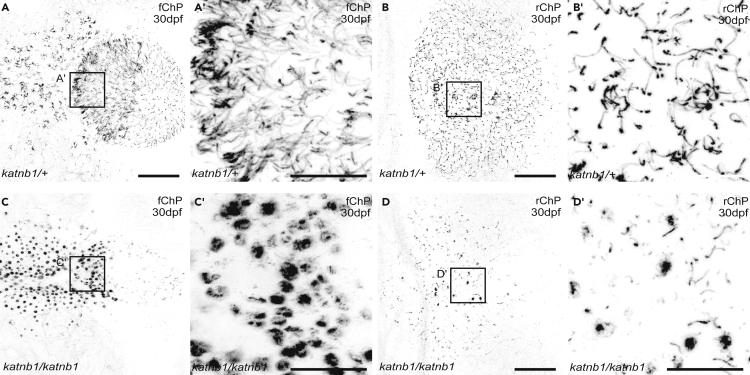
*katnb1* mutants exhibit severe fChP and rChp cilia defects at 30 dpf (A–D) Representative maximum intensity Z-stack projections of confocal micrographs, acquired through dorsally oriented whole mount brains that were dissected from 30 dpf *katnb1*^*mh102/+*^ control (A–B; N = 11, n = 70) and *katnb1*^*mh102/mh102*^ mutant (C-D; N = 11, n = 88) fish, and immunostained for polyglutamylated tubulin. Forebrain ChP (A, C) and rhombencephalic ChP (B, D) are shown. Squares represent higher magnification images of fChP (A′, C′) and rChP (B′, D′). Scale bars, 50 μm (A & B) and 20 μm (A' & B′).

By 30 dpf, both fChP and rChP have developed bundles of multi-cilia in control animals (Figures 5A and 5B), especially on the anterior edge of the fChP and on the dorsal-medial telencephalon, corresponding to the tela choroidea (Nieuwenhuys, 2011; Folgueira et al., 2012; D’Gama et al., 2021). In striking contrast, 30dpf *katnb1*^*mh102/mh102*^ ChPs do not form multi-cilia but instead display circular aggregations of PolyETub staining within anterior fChP and dorsal-medial telencephalic regions (Figures 5C and 5D). In addition, there were significantly fewer and shorter mono-cilia in the rChP of 30dpf *katnb1*^*mh102/mh102*^ mutants compared with controls (Figure S4). Taken together, these observations define a critical period in the ciliation and differentiation of normal zebrafish ChPs, identify defects in *foxj1*-positive ciliated cells that arise just as *katnb1*^*mh102/mh102*^ mutants develop scoliosis, and suggest that abnormal ChP development may contribute to CSF flow defects and spinal curve formation in *katnb1*^*mh102/mh102*^ mutants.

### *katnb1* mutants form a Reissner fiber, despite CSF flow defects

Although observed ChP cilia defects correlate with scoliosis in *katnb1*^*mh102/mh102*^ mutants, they may not be causative. Indeed, scoliosis in *sspo* mutant zebrafish develops in the absence of obvious cilia defects and has instead been linked to defects in RF formation. Furthermore, RF formation defects have also been observed in *ptk7a* and other zebrafish IS models associated with irregularities in motile-cilia-driven CSF flow (Rose et al., 2020; Troutwine et al., 2020), raising the possibility that loss of RF formation may ultimately drive scoliosis in these mutants.

**Figure 6:**
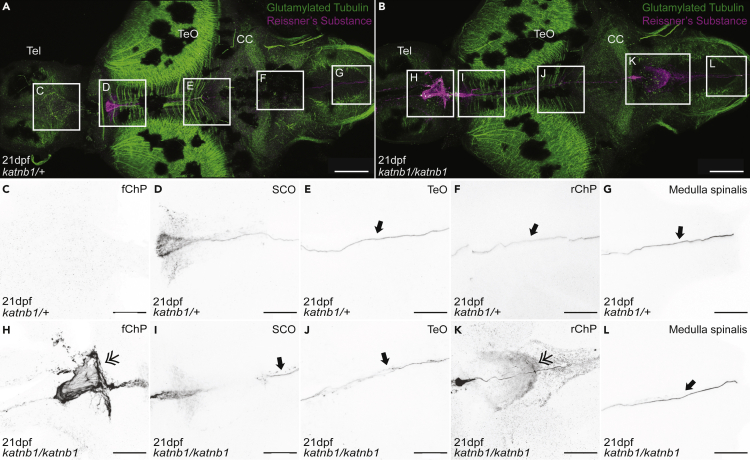
Analysis of Sspo localization and RF formation in 21dpf *katnb1* mutant brains (A–L) Representative maximum intensity Z-stack projections of confocal micrographs, acquired through dorsally oriented whole mount brains that were dissected from 21dpf *katnb1*^*mh102/+*^ control (A, C–G; N = 7, n = 54) and *katnb1*^*mh102/mh102*^ mutant (B, H-L; N = 7, n = 36) fish, and immunostained for polyglutamylated tubulin (green) and Sspo (magenta). Squares indicate regions of interest, chosen for higher magnification analyses of areas surrounding the fChP (C, H); subcommisural organ (SCO; D, I); optic tectum (TeO; E, J); rChP (F, K); and medulla spinalis (G, L). (C–L) Inverted, higher magnification images of Sspo immunostaining. (C–G) Normal Sspo localization is observed in *katnb1*^*mh102/+*^ control animals, including the presence of a RF (arrows). (H–L) *katnb1*^*mh102/mh102*^ mutant images, exhibiting abnormal Sspo accumulation at the fChP and rChP (double arrowheads), and the presence of a RF (arrows). Scale bars, 100 μm (A & B) and 50 μm (C–L).

**Figure 7:**
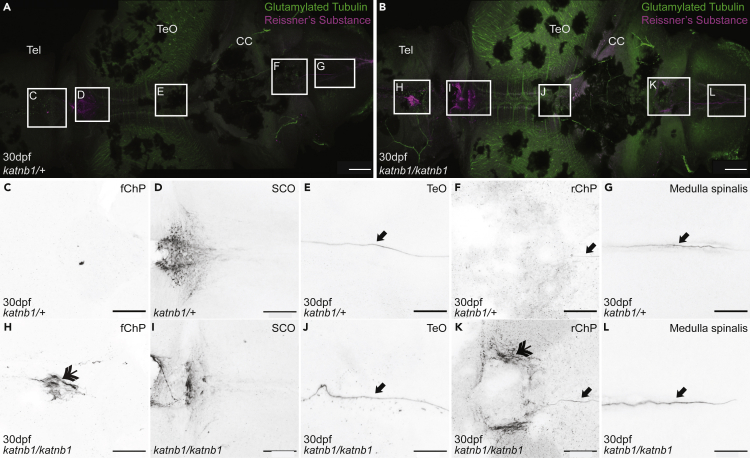
Analysis of Sspo localization and RF formation in 30dpf *katnb1* mutant brains (A–L) Representative maximum intensity Z-stack projections of confocal micrographs, acquired through dorsally oriented whole mount brains that were dissected from 21dpf *katnb1*^*mh102/+*^ control (A, C–G; N = 9, n = 61) and *katnb1*^*mh102/mh102*^ mutant (B, H–L; N = 12, n = 82) fish, and immunostained for polyglutamylated tubulin (green) and Sspo (magenta). Squares indicate regions of interest, chosen for higher magnification analyses of areas surrounding the fChP (C, H); SCO (D, I); TeO (E, J); rChP (F, K); and medulla spinalis (G, L). (C–L) Inverted, higher magnification images of Sspo immunostaining. (C–G) *katnb1*^*mh102/+*^ control animals exhibit normal Sspo localization and RF is present (arrows). (H–L) *katnb1*^*mh102/mh102*^ mutant images, exhibiting abnormal Sspo accumulation at the fChP and rChP (double arrowheads), and the presence of an intact RF (arrows). Scale bars, 100 μm (A & B) and 50 μm (C–L).

To better delineate mechanisms underlying curve formation in *katnb1*^*mh102/mh102*^ mutants, we stained whole brains with an antibody raised against bovine Reissner’s substance that labels zebrafish RF and Sspo (Figures 6 and 7); (Rose et al., 2020). At 21 dpf, a distinct RF was observed initiating in proximity to the SCO and threaded through ventricular cavities of the telencephalon and medulla spinalis of both control (Figures 6A and 6C–6G) and *katnb1*^*mh102/mh102*^ mutant (Figures 6B and 6H–6L) brains. Similar observations were made for 30 dpf samples (Figure 7). However, abnormalities in Reissner’s substance/Sspo distribution were observed in 21 dpf and 30 dpf *katnb1*^*mh102/mh102*^ mutant brains. Specifically, ectopic Sspo aggregation was detected in ventricular cavities underlying both the fChP (Figures 6B, 6H, 7B, and 7H) and rChP (Figures 6B, 6K, 7B, and 7K).

Therefore, CSF flow defects and scoliosis in *katnb1*^*mh102/mh102*^ mutants are not associated with loss of RF formation, albeit may involve decreased functionality or aberrant aggregation of Reissner’s substance. These observations challenge current theories regarding the etiopathogenesis of scoliosis (see Bagnat and Gray, 2020; Ringers and Jurisch-Yaksi, 2020), demonstrating that the presence of RF is not sufficient to keep the spine straight. Rather, the specific and ectopic accumulation of Sspo below the forebrain and rhombencephalic ChPs suggests that the primary defect in *katnb1* IS models relates to abnormal ChP development and/or function with correlative Sspo accumulation subsequent to onset of curvature.

### Cellular stress signatures in *katnb1* mutant bodies and brains

To investigate the molecular consequences of ChP defects, ectopic Sspo accumulation, and disrupted CSF flow on zebrafish brain and spine development, we performed bulk RNA-seq and differential gene expression analysis of dissected 30-dpf brains and 21-dpf trunk/tail segments isolated from scoliotic *katnb1*^*mh102/mh102*^ mutants and their control *katnb1*^*mh102/+*^ siblings. Of note, there was no evidence of dysregulated *urp1* or *urp2* urotensin neuropeptide gene expression in *katnb1*^*mh102/mh102*^ mutants (Tables 1, S1, and S2). This differs from other studies that suggest reduced Urp expression, downstream of abnormal RF-mediated CSF-cN activity, may ultimately drive axial curvature in zebrafish IS models (Böhm et al., 2016; Sternberg et al., 2018; Zhang et al., 2018; Cantaut-Belarif et al., 2020; Lu et al., 2020; Orts-Del’Immagine et al., 2020). Notably, transcriptomic analysis of *sspo*^*dmh4/+*^ mutant brains, which exhibit Sspo secretion and RF formation defects, demonstrated a significant reduction in *urp2* expression (Rose et al., 2020; Table 1). Conversely, bulk RNA-seq analysis of *ptk7a*^*hsc9/hsc9*^ mutant brains, which exhibit ectopic accumulation of RF substance (Rose et al., 2020), demonstrate a significant increase in *urp2* expression (Table 1). Therefore, differential gene expression analysis of dissected brain tissue, which includes the hindbrain/spinal cord junction—an area demonstrated to contain *urp* positive CSF-cNs (Quan et al., 2015) and an intact RF (Rose et al., 2020), can speak to activity of the RF-urp signaling axis. The absence of dysregulated *urp* expression in *katnb1*^*mh102/mh102*^ mutants further suggests that RF assembly/function is normal in this model and provides intriguing evidence that an intact RF-Urp signaling axis is not sufficient to keep the zebrafish spine straight.

**Table 1:** *urp2* expression values from RNA-sequencing of *katnb1*^*mh102/mh102*^, *sspo*^*dmh4*^/+, and *ptk7a*^*hsc9/hsc9*^ mutant animals

Model	*urp2* expression		Data source
	LFC	p-adj	
*katnb1*^*mh102/mh102*^*versus katnb1*^*mh102/+*^ brains	0.3299	0.2976	This study
*sspo*^*dmh4/+*^*versus sspo*^*+/+*^ brains	−1.00387	7.28E-6	Rose et al. (2020)
*ptk7a*^*hsc9/hsc9*^*versus ptk7a*^*hsc9/+*^ heads	1.27966	0.0058	This study

**Figure 8:**
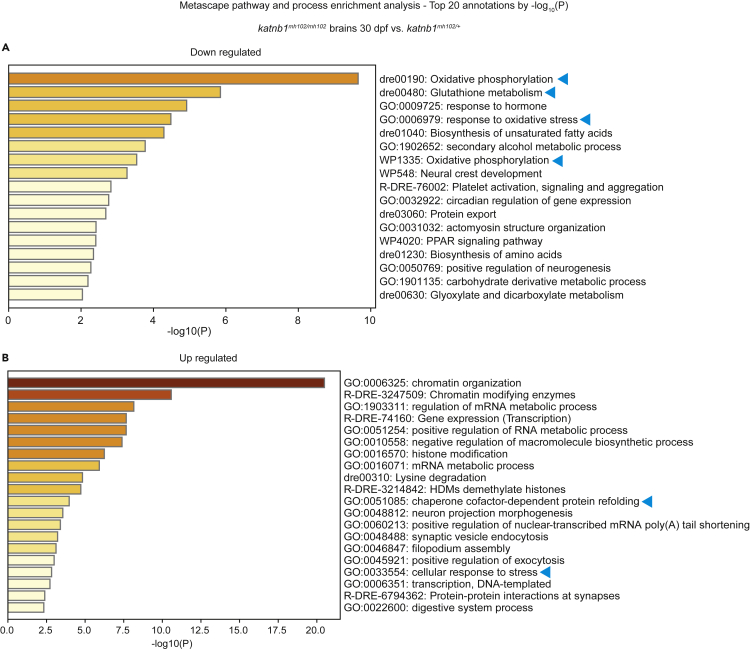
Metascape enrichment analysis of differentially expressed genes in *katnb1* mutant brains (A and B) Metascape pathway enrichment analysis using the *Danio rerio* database for significantly downregulated genes (A) and significantly upregulated genes (B) identified in bulk mRNA sequencing analysis of 30 dpf brains, dissected from *katnb1*^*mh102/mh102*^ mutants compared with *katnb1*^*mh102/+*^sibling controls. Arrows indicate cell-stress response pathways discussed in the text.

As an alternative explanation to the etiopathogenesis of scoliosis, investigations of *ptk7a* and *sspo* IS models implicate neuroinflammatory and oxidative stress responses that arise downstream of CSF homeostasis defects as necessary and sufficient to drive spinal curvature (Van Gennip, Boswell and Ciruna, 2018; Rose et al., 2020). Although 30-dpf *katnb1*^*mh102/mh102*^ mutant brains do not display an obvious inflammatory response at the transcriptional level, there is strong evidence for cell stress: Metascape enriched ontology cluster analyses revealed significant downregulation of pathways involved in oxidative phosphorylation and response to oxidative stress (Figure 8A), and although upregulated pathways were predominantly associated with chromatin remodeling, significant dysregulation of unfolded protein response and cellular response to stress signatures were observed (Figure 8B). Within the trunks and tails of 21-dpf *katnb1*^*mh102/mh102*^ mutants, differential gene expression analyses also identified significant cell stress indicators including upregulated autophagy, downregulated RNA processing, and lipid metabolic processes, in parallel with dysregulated cilia and muscle function signatures (Figures S5A and S5B).

## Discussion

We find that *katnb1*^*mh102/mh102*^ mutant zebrafish exhibit defining attributes of human IS, including juvenile age of onset, scoliosis that progresses in the absence of congenital vertebral malformations, and characteristic patterns of spinal curvature. We demonstrate that Katnb1 function in *foxj1a*-positive motile-ciliated lineages is necessary for normal spine development and when deficient, leads to scoliosis. Further, we show that scoliosis onset in *katnb1*^*mh102/mh102*^ mutants associates with bulk CSF flow defects—a phenotype conserved with *ptk7a* and *sspo* zebrafish IS models (Van Gennip, Boswell and Ciruna, 2018; Rose et al., 2020). Investigation into the timing of EC cilia defects in both *katnb1* and *ptk7a* IS models uncouples EC cilia function from observed phenotypes. Rather, ciliogenesis defects in the developing forebrain, and rhombencephalic ChPs of *katnb1*^*mh102/mh102*^ mutants were found to correlate temporally with CSF flow defects and scoliosis onset, suggesting that the primary deficit in *katnb1* IS models may relate to abnormal ChP development and/or function—a model supported by ectopic aggregations of Sspo protein localized specifically below fChP and rChP in *katnb1*^*mh102/mh102*^ mutants.

Surprisingly, motile-cilia and CSF flow defects do not disrupt RF formation or *urp* gene expression in *katnb1*^*mh102/mh102*^ mutants, providing intriguing evidence that an intact RF-Urp signaling axis is not sufficient to keep the zebrafish spine straight. However, strong cellular and metabolic stress responses were observed in the brains and bodies of scoliotic *katnb1*^*mh102/mh102*^ mutants. Cellular stress can be initiated by multiple factors, including mitochondrial and electron transport chain disruptions and high levels of ROS (Dehdashtian et al., 2018) that can lead to an unfolded protein response and endoplasmic reticulum stress (Xiao and Loscalzo, 2020). Endoplasmic reticulum stress has also been shown to contribute to inflammatory responses and autophagy (Menu et al., 2012; Mou et al., 2020; Thangaraj et al., 2020). These signatures are all present in *katnb1*^*mh102/mh102*^ mutant tissues, although specific cells/tissues of origin remain to be determined. Notably, recent studies have identified neuroinflammatory and oxidative stress signals as being necessary and sufficient to drive spinal curvature downstream of CSF homeostasis defects in *ptk7*a and *sspo* mutant zebrafish (Van Gennip et al., 2018; Rose et al., 2020).

Together with published data on *ptk7a* and *sspo* IS models (Van Gennip et al., 2018: Rose et al., 2020), our data for *katnb1* mutants identify cell stress responses downstream of CSF flow defects as common pathogenic signatures associated with idiopathic-like scoliosis across multiple zebrafish IS models. How these physiological cues ultimately disrupt spine development and their functional relationship to human IS pathogenesis remains to be determined. However, further understanding the role for Katnb1 in ciliogenesis and the functional consequences of *katnb1* mutations on ChP differentiation promise to provide valuable new insights into fundamental mechanisms governing axial morphogenesis.

### Limitations of the study

The functional role for Katnb1 in motile cilia formation and the identity of polyglutamylated structures observed in the ChPs of *katnb1* mutant embryos remain to be determined.

Although *katnb1* mutants demonstrate clear ChP ciliation phenotypes, at this point it is still difficult to fully resolve which *foxj1a*-positive cell types underlie the scoliosis phenotype and whether observed cilia defects in the choroid plexus are causative of the phenotype.

Downstream of *katnb1* motile cilia defects, the specific and causal roles for observed oxidative stress and unfolded protein responses, chromatin/epigenetic dysregulation, autophagy, and metabolic defects in spinal curve formation and progression remain to be determined.

## STAR★Methods

### Key resources table

**Table:** 

REAGENT or RESOURCE	SOURCE	IDENTIFIER
**Antibodies**		

Anti-Reissner’s substance, rabbit polyclonal	(Didier et al., 1995)	Courtesy of Dr. Stephane Gobron
Anti-Polyglutamylation Modification, mouse monoclonal (GT335)	AdipoGen® Life Sciences	Cat# AG-20B-0200-C100; RRID: AB_2490210
Goat anti-Mouse IgG (H + L) Alexa Flour Plus 488	ThermoFisher Scientific	Cat# A32723; RRID: AB_2633275
Goat anti-Rabbit IgG (H + L) Alexa Flour Plus 594	ThermoFisher Scientific	Cat# A32740; RRID: AB_2762824

**Deposited data**		

*katnb1*^*mh102*^ juvenile brain RNAseq dataset	This paper	NIH NCBI: PRJNA870087
*katnb1*^*mh102*^ juvenile trunk/tail RNAseq dataset	This paper	NIH NCBI: PRJNA870759
*ptk7a*^*hsc9*^ juvenile head RNAseq dataset	This paper	NIH NCBI: PRJNA870112
*sspo*^*dmh4*^ juvenile brain RNAseq dataset	(Rose et al., 2020)	NIH NCBI: PRJNA591638

**Experimental models: Organisms/strains**		

*katnb1*^*mh102*^	(Hu et al., 2014)	ZFIN: ZDB-ALT-150311-2
*ptk7a*^*hsc9*^	(Hayes et al., 2013)	ZFIN: ZDB-ALT-130506-1
*Tg(foxj1a*::*katnb1)*	This paper	
*et(cp*:*EGFP)*^*sj2*^	(Henson et al., 2014)	ZFIN: ZDB-ALT-150213-2

**Oligonucleotides**		

Forward sequencing primer for *katnb1*^*mh102*^: 5′-ACACAGACTTCATGTTTCTGACAGGC-3′	(Hu et al., 2014)	
Reverse sequencing primer for *katnb1*^*mh102*^: 5′-TGAGCTCAGACACAACTGAGGGTT-3′	(Hu et al., 2014)	
Forward sequencing primer for *ptk7*^*hsc9*^: 5′-TAATGCAGCCTTATTGTAACGCG-3′	(Hayes et al., 2013)	
Reverse sequencing primer for *ptk7*^*hsc9*^: 5′-AACAGAAAAACACACCATGTCGG-3′	(Hayes et al., 2013)	
Forward Gateway primer of *katnb1*: 5′-GGGGACAAGTTTGTACAAAAAAGCA GGCTTCGCCACCATGGCTCTCACCAACACC-3′	This paper	
Reverse Gateway primer for *katnb1*: 5′-GGGGACCACTTTGTACAAGAAAGCTGGGTG TCAATAGTCCAGAGGGGCC-3′	This paper	

**Software and algorithms**		

Geneious	(Kearse et al., 2012)	https://www.geneious.com/prime/
Fiji	(Schneider et al., 2012; Schindelin et al., 2015)	https://fiji.sc/
Image stitching, ImageJ	(Preibisch et al., 2009)	https://imagej.net/Image_Stitching
GraphPad Prism9	Graph Pad Software	https://www.graphpad.com/scientific-software/prism/
Cutadapt	(Martin, 2011)	https://cutadapt.readthedocs.io/en/stable/
STAR: RNA-seq aligner	(Dobin et al., 2013)	https://github.com/alexdobin/STAR
FastQC	N/A	http://www.bioinformatics.babraham.ac.uk/projects/fastqc/
SAMtools	(Li et al., 2009)	http://www.htslib.org/
RStudio	N/A	http://rstudio.com
Metascape	(Zhou et al., 2019)	https://metascape.org/gp/index.html#/main/step1
Geneious	(Kearse et al., 2012)	https://www.geneious.com/prime/

### Resource availability

#### Lead contact

Further information and requests for resources and reagents should be directed to and will be fulfilled by the lead contact, Brian Ciruna (ciruna@sickkids.ca).

#### Materials availability

There are MTA restrictions to the availability of zebrafish lines generated in this study, due to Institutional policies on the distribution of biological reagents.

### Experimental model and subject details

#### Zebrafish

Established zebrafish husbandry protocols were adhered to and performed in accordance with Canadian Council on Animal Care (CCAC) guidelines. Wild type zebrafish from AB and TU strains were used. Embryos from natural mattings were grown at 28°C. When required, experimental animals were euthanized with tricaine (500 mg/L; MS-222/MESAB), followed by submersion of anesthetized fish in ice water for several minutes. As laboratory zebrafish strains do not utilize a chromosomal sex determination mechanism and sex differentiation does not initiate until after ∼3 weeks post fertilization (Kossack and Draper, 2019), we cannot report sex for our embryonic and juvenile studies. Sex was reported for adult Cobb angle measurements.

### Method details

#### Calcein staining

Staining protocols were followed according to published reports (Du et al., 2001). To prepare Calcein solution, 10 mg of dry calcein powder (Sigma Cas# 154071-48-4) was dissolved in 50 mL of MilliQ water, with pH adjusted to 7.2. Selected fish were transferred to a petri dish filled with above solution. Fish were incubated in dish for 50 min. Solution was removed and fish were transferred to breeding tank filled with system water for 50 min. Animals were anesthetized and imaged on AxioCam MRc 1.4MP Colour Microscope Camera (Zeiss). After sacrificing, larval fish were subsequently genotyped.

#### Microcomputed tomography (μCT)

Fish were fixed in neutral buffered 10% formalin (Sigma) at 4°C overnight and then mounted in 1% low melt agarose (Sigma) in a plastic vial. Specimens were scanned for 4–8 min using SkyScan 1275 high resolution Micro-CT scanner (Bruker) with the X-ray power at 45–50 kV and 200 μA current and reconstructed with 17.74 μm isotropic resolution. 30 dpf samples were scanned for 26 min, Xray power 50 kV, 200 μA current, and 13.69 μm isotropic resolution. The images were analyzed using CTVox software (Bruker), and ImageJ (Schneider et al., 2012; Schindelin et al., 2015). Graphs were generated in Graphpad Prism 9.

#### Scanning electron microscopy (SEM)

Experimental animals were euthanized with tricaine (500 mg/L), followed by submersion of anesthetized fish in ice water for several minutes. Once euthanasia was assured, 3-week, 6-week, and 3-month-old brains were immediately dissected in cold PBST (1× phosphate saline buffer +0.25% TritonX). Whole brains were fixed for 2 h in 2% paraformaldehyde and 2% glutaraldehyde in 0.1M sodium cacodylate buffer (pH7.3), subsequently cut in half and fixed overnight at 4°C. The Nanoscale Bioimaging Facility at PGCRL further prepared samples by rinsing in 0.1M sodium cacodylate buffer with 0.2M sucrose (pH7.3) and brains were gradually dehydrated in an ethanol series (50%, 70%, 90%, 100%). The sagittaly cut brains were critical point dried in a Bal-tec CPD030 critical point dryer, mounted on aluminum stubs, gold coated for 15nm in a Leica ACE200 sputter coater and imaged on a FEI XL30 SEM (Philips).

#### Immunohistochemistry and fixed tissue imaging

Whole mount antibody staining was done on 3- and 4-week-old whole brains. All samples were imaged on an LSM 710 confocal microscope (Zeiss). Images were processed using ImageJ (Preibisch et al., 2009; Schneider et al., 2012; Schindelin et al., 2015) as well as Photoshop (Adobe Creative Cloud).

Juvenile fish were euthanized with tricaine (500 mg/L), followed by submersion of anesthetized fish in ice water for several minutes and immediately dissected in cold PBST (1× Phosphate saline buffer +0.25% Triton X-). Whole brains fixed in 4% Paraformaldehyde (diluted in 1× PBST) at 4°C for at least 24 h. Samples were washed 3 times for 15 min in 1× PBST at room temperature, transferred to 100% Methanol and stored at −20°C for at least 24 h. Samples were rehydrated and washed in 1× PBST, and blocked overnight at 4°C in 10% normal goat serum (ready to use, Life Technologies). Primary antibodies were incubated for 96 h at 4°C in 10% normal goat serum block. The following primary antibodies were used for immunohistochemistry: polyglutamylated tubulin GT335 (1:500, anti-mouse, Adipogen Lifesciences, Cat# AG-20B-0020-C100), GFP polyclonal antibody (1:500, rabbit polyclonal, Thermofisher, A-11122), Reissner’s substance (Didier et al., 1995) (1:500, rabbit polyclonal, gift from Stephane Gobron). Secondary antibodies were incubated for 72 h at 4°C in 10% normal goat serum block. The following secondary antibodies were used for immunohistochemistry: Alexa Fluor Plus 488 (1:1000, goat anti-mouse, Thermofisher, Cat#A32723) and Alexa Fluor Plus 594 (1:1000, goat anti-rabbit, Thermofisher, Cat#A32740). Samples were washed several times for 96 h in 1× PBST between each staining regimen. After last wash, juvenile brain samples were cleared with 100% glycerol, flat mounted using glass slides and coverslipped and imaged.

#### RNA-sequencing

Experimental animals were euthanized with tricaine (500 mg/L), followed by submersion of anesthetized fish in ice water for several minutes. Once euthanasia was confirmed, the head was removed just behind the gill using a scalpel. The brains were then dissected, placed in groups of 3 based on phenotype (severe curvatures (n = 12) and straight siblings (n = 12)) and preserved in RNAlater and RNA Stabilization Reagent (RNeasy Mini Kit, Qiagen) at 4°C. This was repeated with 4 different biological replicates for each genotype. Total RNA extraction was performed using a QIAGEN RNeasy kit following manufacturer’s instructions (Qiagen).

RNA sample quality was assessed using an Agilent Bioanalyzer and 3 samples from each genotype were selected. Library preparation and subsequent sequencing was performed by TCAG at The Hospital for Sick Children (SickKids) using the NEBNext stranded RNA library preparation kit (NEB). Libraries were sequenced on an Illumina NovaSeq 6000 sequencer according to the manufacturer’s instructions, with paired end reads and read lengths 150 bp. Read quality was assessed using FastQC, a standard next generation sequencing quality control tool that evaluates reads per sample, GC content and base quality (Andrews, 2010). Adaptors were trimmed using Cutadapt (Martin, 2011), and reads were reassessed using FastQC. Following this, reads were aligned to the zebrafish genome GRCz10 using STAR (Dobin et al., 2013) and converted to bam files using SamTools (Li et al., 2009). RNA-seq yielded approximately 325–400 million reads per sample.

Read counts and differential gene expression analysis were performed using the SeqMonk program (Andrews, 2007) and Deseq2 Bioconductor R package (Love et al., 2014). All genes with a significance level of p > 0.05 were removed resulting in only significant differentially expressed genes. Metascape (Zhou et al., 2019) was used to further analyze significantly expressed genes by performing enrichment analysis on the set of significantly up-regulated, and down-regulated genes, using the *Danio rerio* datasets.

#### *katnb1*^*mh102/mh102*^ genotyping

The *katnb1*^*mh102/mh102*^ mutant harbors a frameshift mutation in Exon 6 resulting in a truncated protein and loss of function allele (Hu et al., 2014). The genotyping protocol has been described previously (Hu et al., 2014). Genotyping was performed using PCR amplification followed by DNA sequencing submitted to The Center for Applied Genomics (TCAG) at Peter Gilgan Center for Research and Learning (PGCRL). Sequencing results were analyzed using Geneious (Kearse et al., 2012).

#### Live fluorescent dye ventricle injections

Injection protocols have been previously reported (Van Gennip, Boswell and Ciruna, 2018; Rose et al., 2020). Fluorescent dye injections were performed using a microinjection apparatus. 2-week-old fish were anesthetized in a dilute solution of Tricaine. Anesthetized fish were transferred onto an agarose injection plate with anesthetizing agent and system water. Ventricle location was identified using the diamond-shaped pigment pattern on the top of the head, and a glass injection capillary was inserted into that region. Approximately 10 nL of non-toxic violet highlighter ink (Takase et al., 2013) was injected into the ventricle. Injected fish were imaged at 2 h post injection on an Axio ZoomV16 microscope (Zeiss).

#### Transgenesis

Entry plasmids were generated through BP recombination into respective pDONR plasmids (Invitrogen) and then shuttled into standard Tol2 kit Gateway compatible vectors using LR recombination methods to create the final transgenes (Kwan et al., 2007). To generate Tg(floxed-*foxj1a*::*katnb1*) zebrafish, previously generated p5E-*foxj1aP* (Grimes et al., 2016), pME-*katnb1-stop* (extraction of *katnb1* using Gateway primers (detailed below) from a pME18SFL3 eukaryotic expression vector containing *Danio rerio* katanin p80 subunit B1 full length cDNA obtained from the National Institutes of Health Zebrafish Gene Center), and p3E-PolyA were recombined into pDEST Tol2LGSR transgenesis vector. This previously described transgenesis vector is *Cre*-excisable and permits conditional removal of transgenes with a visual readout of lens change colour (Grimes et al., 2016; Van Gennip, Boswell and Ciruna, 2018).

Embryos were injected at the one cell stage with 25 pg of assembled transgene (plasmid) and 25 pg of Tol2 transposase RNA and screened at 48 hpf for transgenesis marker expression. Imaging of reporter expression was performed on an Axio Zoom.V16 (Zeiss). Embryos with strong reporter expression were grown to adulthood and crossed with wildtype fish to establish stable F1 lines. Subsequent F1 lines harboring the transgene were bred into *katnb1*^*mh102/+*^ and maintained in *katnb1*^*mh102/mh102*^ fish.

### Quantification and statistical analysis

#### Cobb angle statistical analysis

Lines were drawn parallel to the top and bottom most displaced vertebrae for each curve. The Cobb angle was then measured as the angle of intersection between lines drawn perpendicular to the original 2 lines (Van Gennip, Boswell and Ciruna, 2018). Analysis was conducted using ImageJ (Schneider et al., 2012; Schindelin et al., 2015). Cobb angle measurements for lateral and dorsal curvatures were summed to obtain a combined Cobb angle measurement for each fish. Results were graphed and statistical significance was calculated using a student’s t-test using GraphPad Prism 9.

#### Choroid plexus cilia quantification

*Et(cp*:*EGFP)*^*sj2*^ fish (Henson et al., 2014) were crossed into *katnb1*^*mh102/+*^ and then offspring were intercrossed to generate heterozygotes and mutants. Brains from these crosses were dissected, fixed, and stained with immunofluorescent antibodies polyglutamylated tubulin GT335 antibody and GFP polyclonal antibody (described above). Brains were imaged on an LSM 710 confocal microscope (Zeiss) at 63×. Images were processed using ImageJ (Preibisch et al., 2009; Schneider et al., 2012; Schindelin et al., 2015) and Z-stack composites were used for analysis. To determine cilia number, a set area of the choroid plexus was used. Cilia in the given area were counted using ImageJ. To determine cilia length, cilia within the above specified area of the ChP were measured using ImageJ. Cilia length for each sample were then averaged. Results were graphed and statistical significance was calculated using a student’s t test using GraphPad Prism9.

## Data Availability

• RNA-seq data have been deposited at NIH NCBI and are publicly available as of the date of publication. Accession numbers are listed in the key resources table. Microscopy data reported in this paper will be shared by the lead contact upon request. • This paper does not report original code. • Any additional information required to reanalyze the data reported in this paper is available from the lead contact upon request.

## Supplemental information

Document S1. Figures S1–S5

Table S1. Differential gene expression analysis of dissected 21-dpf katnb1 mutant trunk/tail segments, related to Figures 8 and S5

Table S2. Differential gene expression analysis of dissected 30-dpf katnb1 mutant brains, related to Figure 8 and Table 1
