# Supplementary material for: Resolving primary pathomechanisms driving idiopathic-like spinal curvature using a new katnb1 scoliosis model
Source: iScience. 2022 Aug 28;25(9):105028. doi: 10.1016/j.isci.2022.105028 (PMC9464966; doi:10.1016/j.isci.2022.105028)
Supplement: Document S1. Figures S1–S5 [file mmc1.pdf]

iScience, Volume *25*

Supplemental information

Resolving primary pathomechanisms driving idiopathic-like spinal curvature using a new *katnb1* scoliosis model

Anne Meyer-Miner, Jenica L.M. Van Gennip, Katrin Henke, Matthew P. Harris, and Brian Ciruna

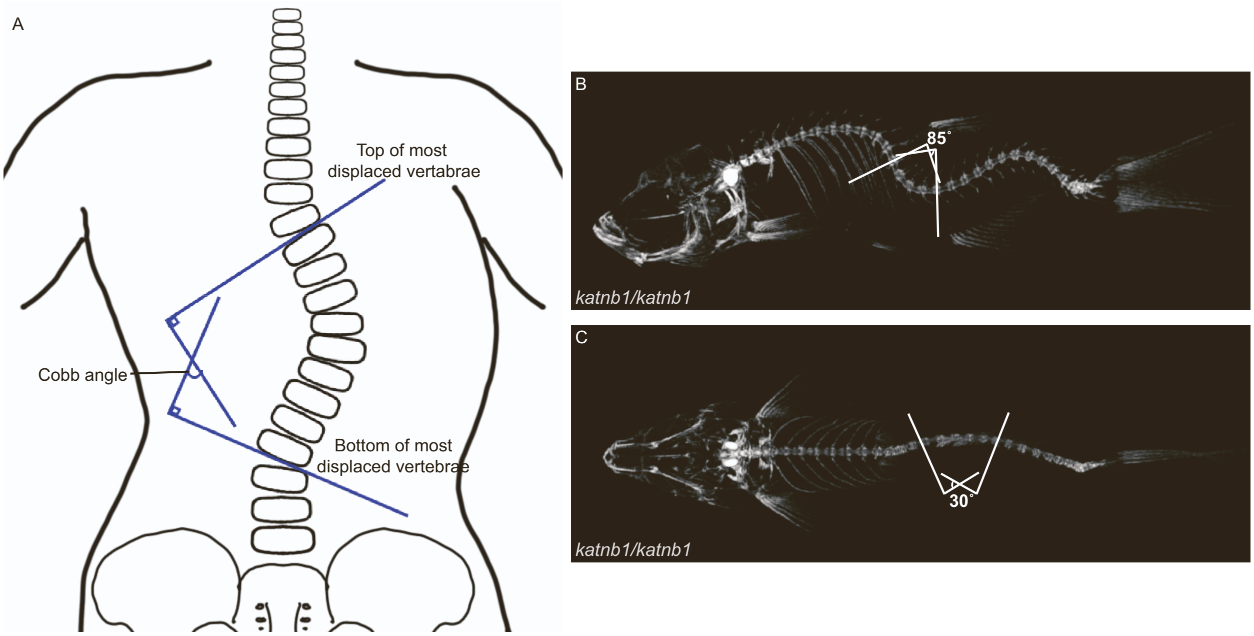

Supplemental Figure 1. Cobb angle measurements to quantify spinal curve severity, Related to Figure 1.

(A) Cobb angle is measured by drawing a line from the top of the most displaced vertebrae, and a line from the bottom most displaced vertebrae of the curvature. Lines are then drawn perpendicular to these two lines, and the angle of intersection between them is defined as the Cobb angle. The larger the curvature, the higher the Cobb angle.
(B) Example of a single Cobb angle in the dorsal-ventral plane of a *katnb1*$^{mh102/mh102}$ microCT image.
(C) Example of a single Cobb angle in the mediolateral plane of a *katnb1*$^{mh102/mh102}$ microCT image.

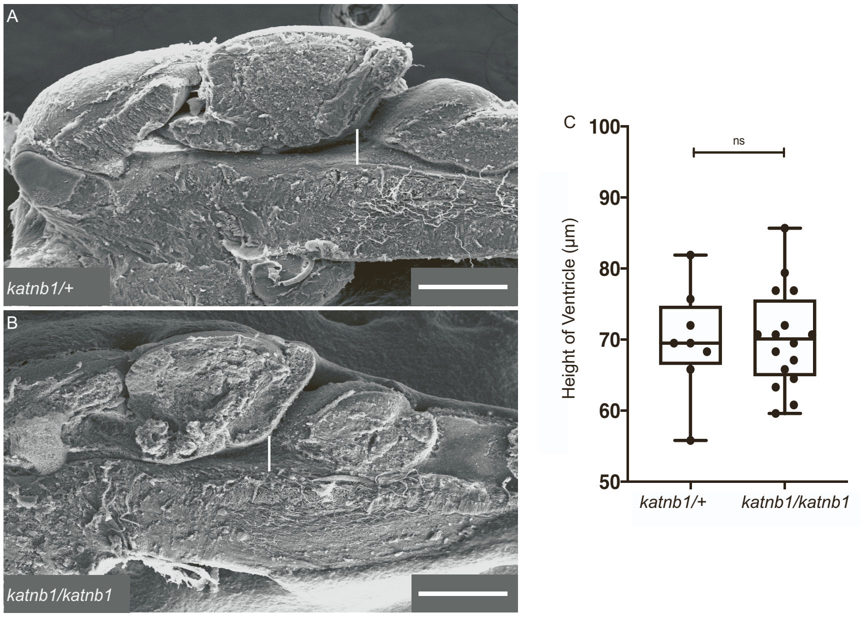

Supplemental Figure 2. *katnb1* mutants do not exhibit hydrocephalus, Related to Figure 2.

(A and **B)** Representative sagittal SEM images of 3-month-old *katnb1*$^{mh102/+}$ (A) and *katnb1*$^{mh102/mh102}$ (B) brains. Scale bars, 200 µm.
(C) Graph quantifies height of ventricle space in micrometers (µm) for *katnb1*$^{mh102/+}$ sibling control (N=3; n=8) and *katnb1*$^{mh102/mh102}$ mutant (N=4, n=16; p=0.9224) brains. Statistical analysis performed using student's t test.

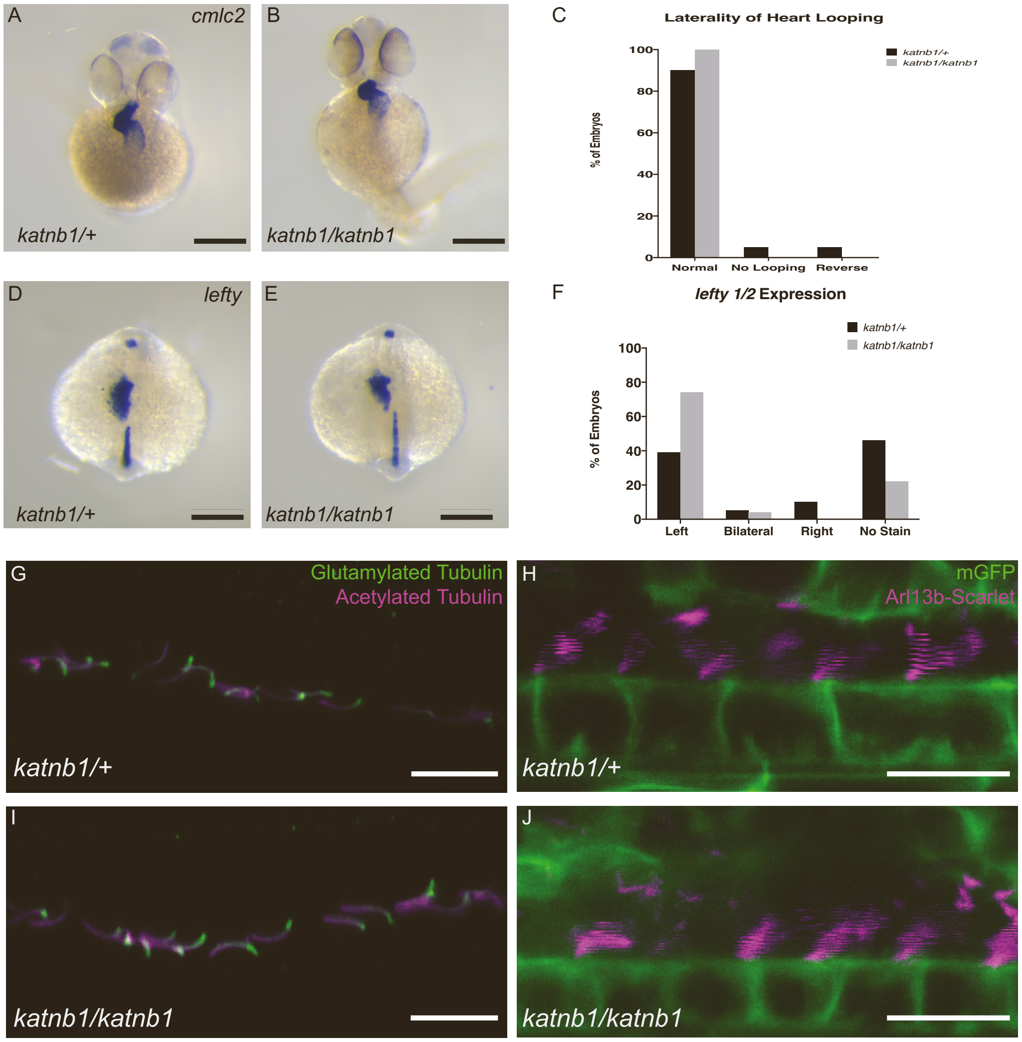

Supplemental Figure 3. Motile cilia are present in embryonic and juvenile *katnb1* mutants, Related to Figures 2 and 3.

(A-C) Whole mount *in situ* hybridization of heart reporter *cmlc2* at 48 hpf in *katnb1*$^{mh102/+}$ (A; N=2, n=22) and *katnb1*$^{mh102/mh102}$ (B; N=2, n=23) embryos reveals normal leftward looping of the developing heart. Graph shows percentage of embryos with normal, reverse and no looping (C). Scale bars, 250 µm.

(D-F) Whole mount in situ hybridization of *lefty1+ lefty2* expression reveals normal left sided gene expression within the lateral plate mesoderm at 20-22 somite-staged *katnb1*$^{mh102/+}$ (D; N=3, n=63) and *katnb1*$^{mh102/mh102}$ (E; N=3, n=22) embryos. Graph shows percentage of embryos with left, bilateral, right or no staining (F). Scale bars, 250 µm.

(G & H) Maximum intensity Z-stack projections of confocal images for laterally mounted 5 dpf *katnb1*$^{mh102/+}$ (G; N=3, n=21) and *katnb1*$^{mh102/mh102}$ (H; N=3, n=10) embryos. Pronephros cilia are immunostained for polyglutamylated tubulin (green) and acetylated tubulin (magenta). Scale bars, 10 µm.

(I & J) Live imaging of cilia lining the floorplate and spinal canal of 30 hpf *katnb1*$^{mh102/+}$ (I; N=3, n=20) and *katnb1*$^{mh102/mh102}$ (J; N=3, n=14) embryos. Membranes labeled with GFP (green) and cilia labeled using arl13b-mScarlet (magenta) reporters. Note the normal orientation and movement (blurred appearance, due to image averaging) of motile floorplate cilia. Scale bars, 10 µm.

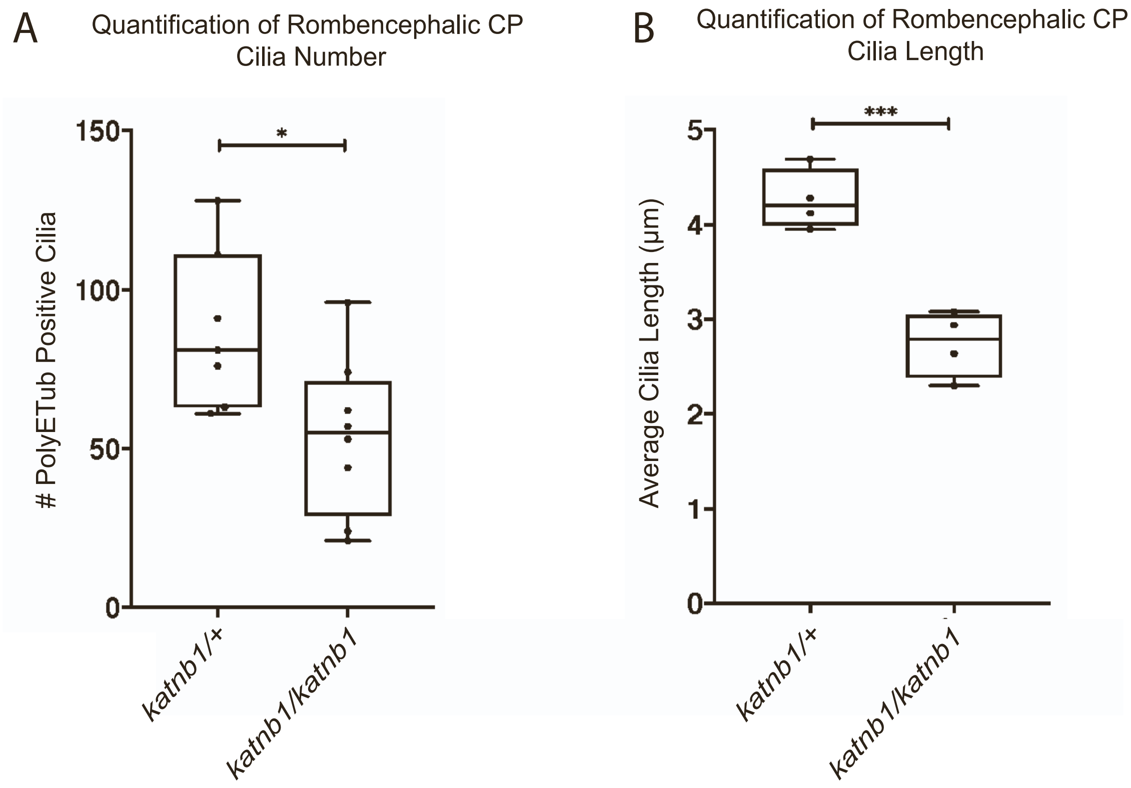

Supplemental Figure 4. Quantification of rChP cilia defects, Related to Figure 5.

(A & B) Graphs show quantification of number of polyglutamylated positive cilia (A; p=0.022) and average length (in µm) of cilia (B; p=0.0006) present in the rhombencephalic choroid plexus (rChP) for 30 dpf *katnb1*$^{mh102/+}$ (N=2, n=8) and *katnb1*$^{mh102/mh102}$ (N=2, n=8) fish. Statistical analysis done using student's t test.

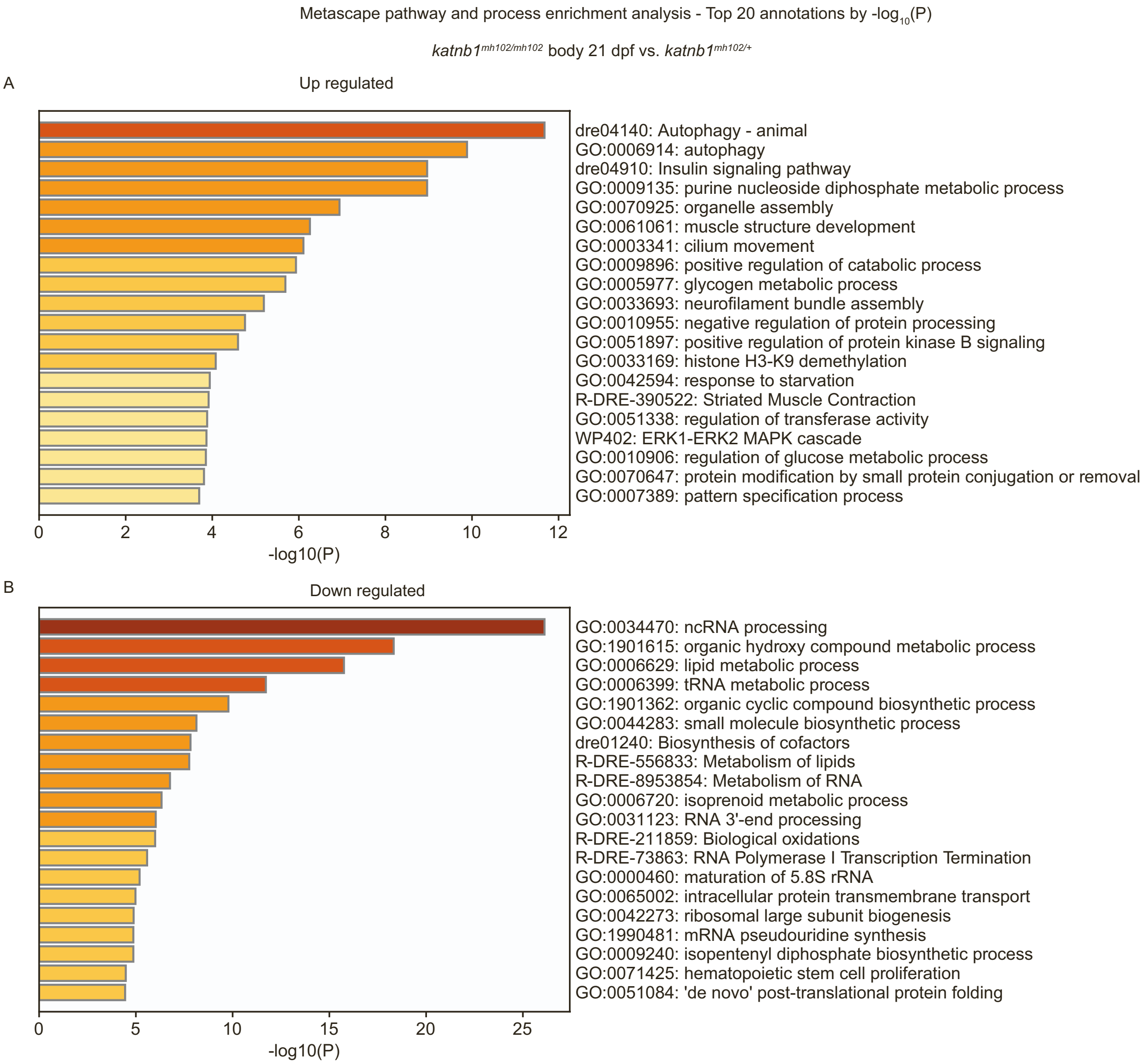

Supplemental Figure 5. Metascape enrichment analysis of differentially expressed genes in *katnb1* mutant trunks/tails, Related to Figure 8.

(A & B) Metascape pathway enrichment analysis using the *Danio rerio* database for significantly down-regulated genes (A) and significantly upregulated genes (B) identified in bulk mRNA sequencing analysis of 21 dpf trunk and tail segments, dissected from *katnb1*$^{mh102/mh102}$ mutants compared to *katnb1*$^{mh102/+}$ sibling controls.
